# Real-World Utilization of Midostaurin in Combination with Intensive Chemotherapy for Patients with *FLT3* Mutated Acute Myeloid Leukemia: A Multicenter Study

**DOI:** 10.3390/jcm15020854

**Published:** 2026-01-21

**Authors:** Sema Seçilmiş, Sibel Kabukçu Hacıoğlu, Fehmi Hindilerden, Burhan Turgut, Düzgün Özatlı, Gülsüm Akgün Çağlıyan, Abdulkadir Baştürk, Aslı Yüksel Öztürkmen, Yavuz Katırcılar, Sinem Namdaroğlu, Başak Ünver Koluman, Cenk Sunu, Serdal Korkmaz, Ayşe Uysal, Yusuf Bilen, Mehmet Ali Erkurt, Mehmet Sinan Dal, Turgay Ulaş, Fevzi Altuntaş

**Affiliations:** 1 Hematology Clinic and Bone Marrow Transplantation Unit, Dr. Abdurrahman Yurtaslan Oncology Education and Research Hospital, University of Health Sciences, 06200 Ankara, Türkiye; dr.sinandal@gmail.com (M.S.D.); turgayulas@yahoo.com (T.U.); faltuntas@hotmail.com (F.A.); 2 Division of Hematology, Pamukkale University, 20160 Denizli, Türkiye; shacioglu@pau.edu.tr (S.K.H.); gcagliyan@pau.edu.tr (G.A.Ç.); bkoluman@pau.edu.tr (B.Ü.K.); 3 Hematology and Apheresis Unit, Bakırköy Dr. Sadi Konuk Education and Research Hospital, University of Health Sciences, 34147 İstanbul, Türkiye; drfehmi_hindi@yahoo.com (F.H.); yagkbln@gmail.com (A.Y.Ö.); 4 Division of Hematology, Tekirdağ Namık Kemal University, 59030 Tekirdağ, Türkiye; bturgut@nku.edu.tr; 5 Division of Hematology, Ondokuz Mayıs University, 55200 Samsun, Türkiye; duzguno@omu.edu.tr; 6 Department of Hematology, Konya City Hospital, University of Health Sciences, 42020 Konya, Türkiye; drbasturk@yandex.com (A.B.); baranserdalkorkmaz@yahoo.com (S.K.); 7 Hematology Clinic and Bone Marrow Transplantation Unit, Kayseri City Hospital, University of Health Sciences, 38080 Kayseri, Türkiye; yavuzktrclr@hotmail.com; 8 Department of Hematology, Dokuz Eylül University, 35210 İzmir, Türkiye; sinem.namdaroglu@deu.edu.tr; 9 Department of Hematology, Sakarya University, 54187 Sakarya, Türkiye; csunu@sakarya.edu.tr; 10 Division of Hematology, Fırat University, 23119 Elazığ, Türkiye; drayseorucuysal@gmail.com; 11 Value Added Medicine Medicalpark Bursa Hospital, İstinye University, 16220, Bursa, Türkiye; bilenyusuf@hotmail.com; 12 Division of Hematology, İnönü University, 44280 Malatya, Türkiye; erkurtali@hotmail.com; 13 Division of Hematology, Department of Internal Medicine, School of Medicine, Yıldırım Beyazıt University, 06800 Ankara, Türkiye

**Keywords:** acute myeloid leukemia, AML, *FLT3* inhibitors, midostaurin

## Abstract

**Background/Objectives:** Real-world data on the therapeutic use of FLT3 inhibitors in Turkey remain limited. Therefore, we retrospectively evaluated outcomes from 13 academic centers nationwide, focusing on the multikinase inhibitor midostaurin in patients with newly diagnosed FLT3-mutated acute myeloid leukemia (AML). **Methods:** We collected comprehensive information regarding treatment efficacy, safety, and tolerability. **Results:** The overall response rate to intensive chemotherapy (3 + 7) plus midostaurin was 87.7%, with a complete remission rate of 84.2%, consistent with previously reported clinical trial results. Treatment discontinuation due to intolerance or toxicity was low (3.5%). One patient discontinued therapy because of septic shock during induction, and another due to a drug–drug interaction during consolidation. Median overall survival was 21.4 months. Allogeneic stem cell transplantation was performed in first remission in 52.6% of patients. Five patients (8.8%) were refractory to induction therapy, and relapse occurred in 21.1% (12 patients). **Conclusions:** These findings support the effectiveness and acceptable tolerability of midostaurin in routine clinical practice for FLT3-mutated AML.

## 1. Introduction

Acute myeloid leukemia (AML) is a group of hematopoietic progenitor stem cell cancers that exhibit varying clinical outcomes due to their cytogenetic and molecular diversity [1]. AML occurs more frequently in males (male–female ratio: 1.5) [2]. The median age at diagnosis exceeds 65 years, and its incidence increases progressively with age [3].

Despite improvements in the treatment of AML, the risk of relapse remains significant. This risk is influenced by the patient’s age and the genetic traits of the leukemia. The five-year relative survival rate for AML is around 35%, but this rate declines to less than 10% for patients older than 65 [4]. Prognostic risk in AML is determined at the time of diagnosis based on specific cytogenetic and molecular abnormalities. Our understanding of the molecular foundations of AML continues to expand. Genetic alterations in AML are recurrent and include amplifications, deletions, rearrangements, and point mutations [1]. These findings guide treatment decisions.

The Fms-like tyrosine kinase 3 gene (*FLT3*) is a receptor tyrosine kinase expressed by hematopoietic stem and progenitor cells, playing a crucial role in the early stages of developing myeloid and lymphoid lineages. *FLT3* mutations disrupt the physiological balance between proliferation and differentiation, leading to overexpression or constitutive activation of the receptor, thereby driving leukemic cell expansion and supporting AML blast survival [5].

Mutations in the *FLT3* gene are found in approximately 30% of all AML cases and are particularly common in patients with a normal karyotype. There are two subtypes of *FLT3* mutations, with the most prevalent being the internal tandem duplication (*ITD*), which accounts for 25% of all AML cases. *FLT3-ITD* is a driver mutation associated with high leukemic burden, poor prognosis, and increased relapse risk. *ITD* mutations induce constitutive activation of the *FLT3* signaling pathway, promoting leukemic proliferation and therapy resistance. The second subtype, *FLT3* tyrosine kinase domain (*TKD*) mutations, occurs in 7–10% of AML cases. Its prognostic significance remains uncertain. Furthermore, *FLT3* mutational status is dynamic and may change during the disease course, reinforcing the importance of assessing clonal evolution at diagnosis and relapse [6].

Given the adverse prognostic impact of *FLT3* mutations, several small-molecule *FLT3* inhibitors have been developed to improve outcomes when combined with intensive chemotherapy. Recent evidence demonstrates that incorporating *FLT3* inhibition into frontline AML therapy decreases relapse and improves overall survival, particularly with midostaurin [7]. These findings reinforce the role of *FLT3* inhibitors as an important component of intensive induction and consolidation regimens.

Midostaurin is the first *FLT3* inhibitor approved for treating adult patients with newly diagnosed AML with *FLT3* mutations. It is used in combination with standard induction therapy consisting of cytarabine and daunorubicin, as well as cytarabine for consolidation therapy. Adding midostaurin to the standard intensive chemotherapy regimen [8] was found to improve median event-free survival (EFS) significantly (*p* = 0.009) and median overall survival (OS) (*p* = 0.002) [9,10]. This approval is based on the results of the pivotal phase 3 study known as RATIFY [11]. In the RATIFY study, midostaurin was shown to enhance overall survival (OS) in all types of *FLT3* mutations, including those with *TKD*, low allelic ratio *ITD*, and high allelic ratio *ITD*, regardless of the *FLT3-ITD* allelic ratio [10].

The RATIFY study included de novo AML patients aged 18–59; however, midostaurin has received approval for use across all age groups and for newly diagnosed AML patients, including those with secondary AML. In the German–Austrian Acute Myeloid Leukemia Study Group (AMLSG) 16–10 phase 2 clinical trial, midostaurin was demonstrated to be safe and effective, leading to a significant improvement in outcomes for *FLT3-ITD* positive AML patients aged 60 to 70 years, as well as for younger patients, compared to historical controls [12].

Currently, data regarding the safety and efficacy of midostaurin with an idarubisin-based 7 + 3 AML induction regimen and consolidation therapy with cytarabine in AML populations remain limited. In this study, we conducted a multicenter retrospective analysis of our experience using midostaurin in induction and consolidation therapy. We specifically evaluated the effects and toxicity of adding midostaurin, a multikinase *FLT3* inhibitor, to standard chemotherapy in AML patients with *FLT3* mutations.

## 2. Materials and Methods

The study was carried out with the permission of Ankara Dr. Abdurrahman Yurtaslan, Oncology Training and Research Hospital Ethics Committee, on 26 January 2022, with decision number 2022-01/1608. All procedures strictly adhered to the principles outlined in the 1964 Helsinki Declaration, ensuring the ethical conduct of the study. Each patient provided written informed consent prior to participation. Each participating center obtained local ethics approval in accordance with institutional requirements for retrospective data collection.

### 2.1. Patients and Study Design

This retrospective observational study focused on patients with AML who were newly diagnosed with *FLT3* mutations (*ITD* and/or *TKD*) through molecular analysis. Eligible patients had not received prior antineoplastic therapy other than hydroxyurea and/or cytarabine for cytoreduction. The study was conducted at 13 academic centers in Turkey between September 2019 and May 2022.

This research included *FLT3*-positive AML patients aged between 18 and 70 years, with an ECOG performance score of 0 to 2, who were deemed eligible for intensive chemotherapy. Acute promyelocytic leukemia patients were excluded. Patients with significant comorbidities accompanying AML and those with bilirubin levels exceeding 2.5 times the upper limit of the normal range were also excluded.

*FLT3* mutation assessment was performed using PCR-based fragment analysis for the identification of both *ITD* and *TKD* mutations, according to each center’s standard molecular diagnostic procedures. Allelic ratio measurement was not consistently available across all centers and was therefore not incorporated into the analysis.

These patients received intensive induction chemotherapy in combination with midostaurin, followed by consolidation therapy using cytarabine and midostaurin. Data were collected manually from medical records. All centers used a unified case report form to ensure standardized data extraction, including demographics, laboratory values, molecular findings, treatment details, toxicities, and clinical outcomes. Missing data were handled through complete-case analysis. The institutional review board at each participating center reviewed and approved the trial protocol.

### 2.2. Treatment Protocol

The same chemotherapy doses and transplantation protocols were consistently utilized across all participating centers throughout the study. The 3 + 7 induction therapy consisted of 12 mg/m^2^ of idarubicin, administered via rapid intravenous injection on days 1, 2, and 3, along with 100 mg/m^2^ of cytarabine, given as a continuous intravenous infusion from days 1 to 7. A bone marrow examination was conducted around day 30 to evaluate remission status.

Patients who achieved complete remission after induction therapy received either medium-dose (1.5 to 2 g/m^2^) or high-dose (3 g/m^2^) cytarabine, with dose reductions at the physician’s discretion based on age and/or comorbidities. This cytarabine was administered as a 3-h infusion every 12 h on days 1, 3, and 5 for a total of 1 to 4 cycles, within a 28-day cycle.

Furthermore, midostaurin was given orally at a dose of 50 mg twice daily for 14 days, starting on the eighth day after initiating induction and consolidation chemotherapy. Midostaurin was not administered if a patient’s corrected QT interval exceeded 500 ms or if they experienced a grade 3 or 4 adverse effect related to midostaurin. Any missed doses of midostaurin were not readministered.

Across all centers, anti-infective prophylaxis (including antibacterial, antiviral, and antifungal agents) was administered according to institutional standards, and all patients received transfusion support based on routine hematologic thresholds. Tumor lysis syndrome prophylaxis (hydration and allopurinol or rasburicase) was provided before induction chemotherapy. Growth factor support (G-CSF) during post-induction marrow aplasia was used at the discretion of the treating physician. Electrocardiographic monitoring was routinely performed during midostaurin administration to assess QTc intervals.

During induction, patients received inpatient care with daily monitoring of complete blood counts, renal and hepatic function tests, and infection parameters. Microbiological cultures, imaging studies, and empirical antibiotic escalation followed center-specific febrile neutropenia guidelines. Similarly, patients were also hospitalized during consolidation therapy, with daily monitoring of laboratory values, toxicity assessments, and infection surveillance in accordance with institutional supportive-care protocols.

The decision to perform allogeneic stem cell transplantation and the timing of the procedure were left to the treating physician’s discretion. Patients who were eligible for transplantation and had an available donor were referred for the procedure during the consolidation treatment process. Institutional policies ensured supportive care throughout therapy. Transplant eligibility evaluation included donor availability, remission status, comorbidity assessment, and institutional transplant board approval. Conditioning regimens (myeloablative or reduced-intensity) and graft-versus-host disease prophylaxis protocols were selected according to the standard practices of each participating center.

### 2.3. Study Outcomes

The primary endpoint was median overall survival (OS), which was defined as the time from start of induction therapy to death from any cause. Overall response rate (ORR) is defined as the proportion of patients who achieved either a complete remission (CR) or complete remission with incomplete count recovery (CRi). CR was determined by bone marrow blasts < 5% by morphological evaluation, an absolute neutrophil count (ANC) > 1000 cells/μL, and platelet count >100,000/μL (in addition to the absence of circulating blasts, Auer rod-containing blasts, and extramedullary disease). Patients with CRi met all CR criteria except with residual neutropenia (ANC < 1000 cells/μL) or thrombocytopenia (<100,000/μL). Treatment response was evaluated according to standard, internationally accepted criteria for acute myeloid leukemia.

Secondary endpoints were the 30-day mortality rate, the rate of documented infections, and the incidence of adverse events during both induction and consolidation phases. Infections were categorized based on clinical presentation, microbiological identification, or radiologic confirmation. Relapse assessments were recorded according to standard institutional timelines, while bone marrow evaluations for remission assessment were uniformly performed around day 30. Induction mortality was defined as death occurring within the first 30 days of treatment.

Additionally, we analyzed disease- and patient-specific parameters and the toxicity and efficacy results. Toxicity was classified based on the Common Terminology Criteria for Adverse Events (CTCAE) version 4.03 [13], and adverse events were categorized as “any grade” and “grade 3–4” toxicities in accordance with this classification.

### 2.4. Statistical Analysis

Patient characteristics were summarized using frequencies (numbers and percentages) for categorical variables and median and range for continuous variables. Kaplan-Meier analysis was used for survival analysis. Less than 0.05 was considered statistically significant for all analyses. All statistical analyses were performed with the SPSS software package (Version 26, IBM, Armonk, NY, USA).

## 3. Results

A total of 57 patients were included in the analysis, with those who survived having a median follow-up period of 15.6 months (ranging from 0.9 to 31.6 months). The median age of the patients was 55 years, ranging from 20 to 70. Among the patients, 31.5% were over the age of 60, and 3.5% were over 70. *FLT3-ITD* mutations were found in 48 patients, accounting for 84.2% of the group, while *FLT3/TKD* mutations were identified in 7 patients, representing 12.3%. Additionally, two patients (3.5%) had both *TKD* and *ITD* mutations. The majority of patients (86%) had de novo AML. Of the samples studied, 82.5% displayed a normal karyotype, while 21.1% exhibited *NPM1* mutations. Details of the patients’ clinical and demographic characteristics are given in Table 1.

Most patients completed the full 14-day course of midostaurin during the induction phase. Treatment was discontinued for two patients (3.5%) due to severe side effects. One patient discontinued treatment due to septic shock during induction therapy, while another patient stopped treatment because of a drug-drug interaction during consolidation therapy. In four patients (7%), dose reductions or treatment interruptions occurred at various stages of therapy. This included two patients who experienced severe sepsis, one patient with a prolonged QT interval, and one patient with severe refractory thrombocytopenia and purpura. See Table 2.

**Figure 1:**
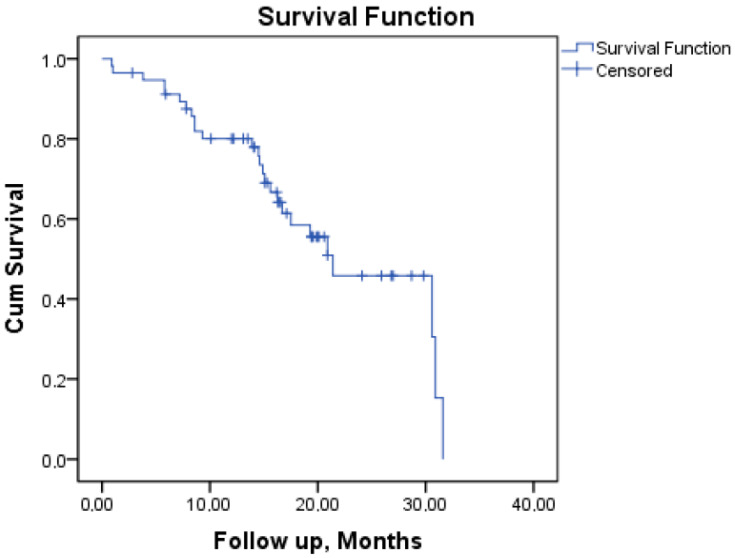
Kaplan–Meier overall survival (OS) curve.

Forty-eight patients (84.2%) achieved CR and two (3.5%) achieved CRi. Death during induction was reported in 2 (3.5%) patients (due to sepsis). Five patients (8.8%) did not respond to the induction therapy. Additionally, relapse was observed in 21.1% of the cases, affecting 12 patients. Median OS was 21.4 months (95% confidence interval [CI]) (See Figure 1). Transplantation was performed at some point during the disease course in 54.4% of the patients (31 patients); it was performed during the first complete remission in 52.6% of the patients. Survival and treatment response outcomes for the overall cohort are seen in Table 3.

The rate of grade 3 or 4 neutropenia, lymphopenia, thrombocytopenia, and anemia was 80.7%, 71.9%, 84.2%, and 68.4%, respectively. Grade 3/4 diarrhea, vomiting, and hypocalcemia were reported in 5.2% of patients each. Grade 3/4 fatigue occurred in 10.5% of patients, while grade 3/4 nausea was seen in 14%. Grade 3/4 hypokalemia was observed in 26.3%, and all these side effects were manageable. Additionally, pneumonitis or pulmonary infiltrates were noted in 43.8% of patients, rectitis in 12.2%, rash in 24.5%, and sepsis in 7%. Other common adverse events during the induction and consolidation courses are summarized in Table 4.

## 4. Discussion

This multicenter retrospective study evaluated the real-world effectiveness and safety of combining intensive 3 + 7 induction chemotherapy with midostaurin, followed by consolidation therapy, in patients with *FLT3*-mutated AML treated across 13 centers in Turkey between 2019 and 2022. Our findings contribute to the growing body of evidence supporting *FLT3* inhibition in routine clinical practice and offer insight into patient groups underrepresented in pivotal trials.

AML is most commonly diagnosed in older adults, with a median age of 68 years [8]. In contrast, the RATIFY trial included a younger population (median age 47 years; range 18–59) [9] and excluded both patients ≥60 years and those with secondary AML. Although midostaurin has no age-based restrictions, its use in older adults requires careful assessment of comorbidities and fitness for intensive therapy [14]. In a multicenter cohort of patients ≥60 years with newly diagnosed *FLT3*-mutated AML, midostaurin combined with intensive chemotherapy demonstrated acceptable efficacy [15]. Our study extends these observations by including a substantial proportion of older adults (31.3%) and secondary AML cases, reflecting a more heterogeneous real-world population.

Patients in our study were older overall (median age 55, range 20–70) and had a higher frequency of normal karyotype (82.5%). Baseline leukemic burden was also greater, with a median WBC count of 43,990/μL—approximately 9000 cells/μL higher than in the RATIFY cohort. Moreover, the prevalence of *FLT3-ITD* was higher in our population (84.2%), while *FLT3-TKD* mutations were less common (12.3%) compared to RATIFY (77.5% *ITD*; 22.5% *TKD*).

Despite these higher-risk characteristics, the real-world CR rate in our study was 84.2%, markedly higher than the 58.9% reported in the RATIFY trial. This finding is notable given the inclusion of older adults, a higher proportion of *FLT3-ITD*, and patients with secondary AML. The absence of allelic ratio assessment may have influenced the interpretation of *FLT3-ITD* risk. Additionally, because midostaurin is a multikinase inhibitor, inhibition of other oncogenic pathways may have contributed to improved response rates. Similar favorable outcomes have been reported in other real-world studies evaluating *FLT3* inhibitors during induction therapy [16,17,18,19].

Early pharmacovigilance studies identified anemia, rash, nausea, vomiting, and fever as common grade ≥3 events with midostaurin. An FDA Adverse Event Reporting System analysis recorded 5938 midostaurin-related adverse events reported from 2015–2022, with sepsis, pneumonia, and diarrhea being the events most strongly associated with mortality [20]. European Medicines Agency (EMA) reviews similarly identified febrile neutropenia, nausea, exfoliative dermatitis, vomiting, headache, petechiae, and fever as the most frequent adverse reactions [21].

In our study, toxicity patterns largely mirrored expectations for intensive AML therapy. The most common grade 3–4 adverse events were cytopenias and infections, consistent with the RATIFY trial, where febrile neutropenia occurred in 82% and infections in 52% of patients. We observed febrile neutropenia in 82.5% of patients, nearly identical to RATIFY, and the predominant treatment-related deaths were infection-related. Unlike RATIFY, however, no grade ≥3 rash was observed in our cohort. Overall, toxicity profiles remained manageable within standard supportive-care protocols.

Allogeneic stem cell transplantation (allo-SCT) in first CR improves long-term outcomes for patients with *FLT3-ITD* AML. RATIFY demonstrated that allo-SCT in CR1 was associated with favorable survival outcomes [22].

In RATIFY, 28.1% of patients receiving midostaurin proceeded to allo-SCT in CR1. In our real-world cohort, CR1 transplantation was substantially higher (52.6%), and 54.4% underwent transplantation at any time during the disease course, reflecting national practice patterns and donor availability.

Prior studies have suggested that *NPM1/FLT3-ITD* genotypes, WBC count at diagnosis, and allo-SCT are independent prognostic factors for OS [23]. In contrast, our study found no significant impact of *NPM1* mutation status, *FLT3-ITD*, *FLT3-TKD*, WBC count, or transplantation on OS. This may be related to sample size limitations, heterogeneity across centers, lack of allelic ratio data, and relatively short follow-up, emphasizing the need for larger prospective studies.

Overall, this study provides additional real-world evidence supporting the use of midostaurin in *FLT3*-mutated AML. Our findings confirm high remission rates, manageable toxicity, and transplantation feasibility in routine practice, consistent with clinical trial data. However, larger comparative trials incorporating molecular risk stratification, allelic ratio evaluation, and long-term survival endpoints are needed to refine prognostic interpretation and optimize treatment strategies for this high-risk patient population.

## 5. Conclusions

This study demonstrates that oral midostaurin, administered at 50 mg twice daily for 14 days beginning on day 8 of induction and consolidation therapy, can be used safely and effectively in patients with *FLT3*-mutated AML. The regimen showed high remission rates, an acceptable toxicity profile, and remained feasible even in older patients and those with secondary AML. These findings support the continued adoption of *FLT3* inhibition in real-world clinical practice, while highlighting the need for larger prospective studies to further clarify prognostic factors and long-term outcomes.

## Figures and Tables

**Table 1:** Clinical and demographic characteristics of 57 AML patients.

Characteristics	
Gender,	*n* (%)
Female	28 (49.1)
Male	29 (50.9)
Age at AML diagnosis, years (range)	5 (20–70)
≥60 years old at AML diagnosis	18 (31.5)
ECOG	
0	16 (28.1)
1	28 (49.1)
2	13 (22.8)
Comorbidities	
DM	8 (14)
HT	20 (35.1)
CKD	5 (8.8)
COPD	6 (10.5)
CAD	6 (10.5)
Breast cancer	1 (1.8)
Hypothyroidism	3 (5.3)
Disease characteristics	
2017 ELN risk stratification by genetics	*n* (%)
Favourable	2 (3.5)
Intermediate	23(40.3)
Adverse	32 (56.1)
Karyotype, *n* (%)	
Normal karyotype	47 (82.5)
Abnormal karyotype	10 (17.5)
Subtype of *FLT3* mutation—*n* (%)	
*ITD*	48 (84.2)
*TKD*	7 (12.3)
Both	2 (3.5)
*NPM1* mutated, n (%)	12 (21.1)
Leukemia, n (%)	
De novo	49 (86)
Secondary	8 (14)
Clinical and laboratory characteristics at presentation	
Median white blood count per μL (range)	43,990 (340–361,850)
Median platelet count per μL (range)	56,000 (6000–191,000)
Median hemoglobin level (range) (g/dL)	8 (5–13)
Median % marrow blasts	80 (20–98)

DM: Diabetes mellitus, HT: Hypertension, CKD: Chronic kidney disease, COPD: chronic obstructive pulmonary disease, CAD: Coronary artery disease.

**Table 2:** Treatment characteristics.

Variables	*n* (%)
Number of patients that received consolidation chemotherapy	50 (87.7)
Median number (range) of consolidation cycles	3 (0–4)
HSC transplantation	31 (54.4)
HSC transplantation in CR1	30 (52.6)
Midostaurin treatment	
During induction	
Number of patients with stopped treatment	1 (1.7)
Number of patients with dose reduction/interruption	2 (3.5)
During consolidation	
Number of patients with stopped treatment	1 (1.7)
Number of patients with dose reduction/interruption	2 (3.5)

**Abbreviations:** HSC, hematopoietic stem cell; CR1, first complete remission.

**Table 3:** Survival and treatment response outcomes for the overall cohort.

Outcome	Definition	n (%)
OS	Alive	31 (54.4)
	Dead	26 (45.6)
	Median OS (months)	21.4
Allogeneic HSCT	HSCT received	31 (54.4)
	Time to HSCT (months)	4.2 (2–16.4)
ORR rate		
CR	48 (84.2)	
Cri	2 (3.5)	
Refractory	5 (8.8)	
Induction death	2 (3.5)	

**Abbreviations:** OS, overall survival; HSCT, hematopoietic stem cell transplantation; ORR, overall response rate; CR, complete remission; CRi, complete remission with incomplete hematologic recovery.

**Table 4:** Summary of frequent adverse events during induction and consolidation courses.

Variables	Any Grade	Grades 3–4
Hematological adverse events, *n* (%)		
Neutropenic fever episodes, *n* (%)	47 (82.5)	47 (82.5)
Leukopenia	51 (89.4)	44 (77.1)
Neutropenia	53 (92.9)	46 (80.7)
Lymphopenia	50 (87.7)	41 (71.9)
Thrombocytopenia	52 (91.2)	48 (84.2)
Anemia	53 (92.9)	39 (68.4)
Non-hematological adverse events, *n* (%)		
Rash	14 (24.5)	0
Gastrointestinal toxicity		
Diarrhea	21 (36.8)	3 (5.2)
Nausea	56 (98.2)	8 (14)
Vomiting	50 (87.7)	3 (5.2)
Constipation	24 (42.1)	0
Hepatic toxicity		
Hyperbilirubinemia	15 (26.3)	1 (1.7)
Increased alanine aminotransferase	19 (33.3)	2 (3.5)
Increased aspartate aminotransferase	15 (26.3)	0
Renal toxicity		
Hypokalemia	39 (68.4)	15 (26.3)
Hyponatremia	13 (22.8)	1 (1.7)
Hypophosphatemia	14 (24.5)	0
Hypocalcemia	19 (33.3)	3 (5.2)
Infection		
Pneumonitis or pulmonary infiltrates	25 (43.8)	
Coronavirus disease (COVID-19)	2 (3.5)	
Rectitis	7 (12.2)	
Sepsis or septic shock	4 (7)	
Other infections	15 (26.3)	
Pain	29 (50.8)	2 (3.5)
Fatigue	54 (94.7)	6 (10.5)
Myocardial infarction	1 (1.7)	
QT prolongation	1 (1.7)	

**Abbreviations:** QT, QT interval.

## Data Availability

Data are available upon request.

## Abbreviations

AML, acute myeloid leukemia; ECOG, Eastern Cooperative Oncology Group; DM, diabetes mellitus; HT, hypertension; CKD, chronic kidney disease; COPD, chronic obstructive pulmonary disease; CAD, coronary artery disease; ELN, European LeukemiaNet; *FLT3*, FMS-like tyrosine kinase 3; *ITD*, internal tandem duplication; *TKD*, tyrosine kinase domain; *NPM1*, nucleophosmin 1.
